# Low Silver/Copper Exchange in a Copper-Phosphate Enzyme Nanoflower Hybrid Extremely Enhanced Antimicrobial Efficacy against Multidrug Resistant Bacteria

**DOI:** 10.1021/acsabm.4c00898

**Published:** 2024-09-10

**Authors:** Clara Ortega-Nieto, Noelia Losada-Garcia, Pilar Domingo-Calap, Miroslawa Pawlyta, Jose M. Palomo

**Affiliations:** † Instituto de Catálisis y Petroleoquímica (ICP), CSIC, 28049 Madrid, Spain; ‡ Institute for Integrative Systems Biology (I2SysBio), Universitat de València-CSIC, 46980 Paterna, Spain; § Materials Research Laboratory, Faculty of Mechanical Engineering, Silesian University of Technology, Konarskiego 18A, 44-100 Gliwice, Poland

**Keywords:** silver, nanoparticles, enzyme−metal biohybrids, antimicrobial activity, multidrug resistant bacteria

## Abstract

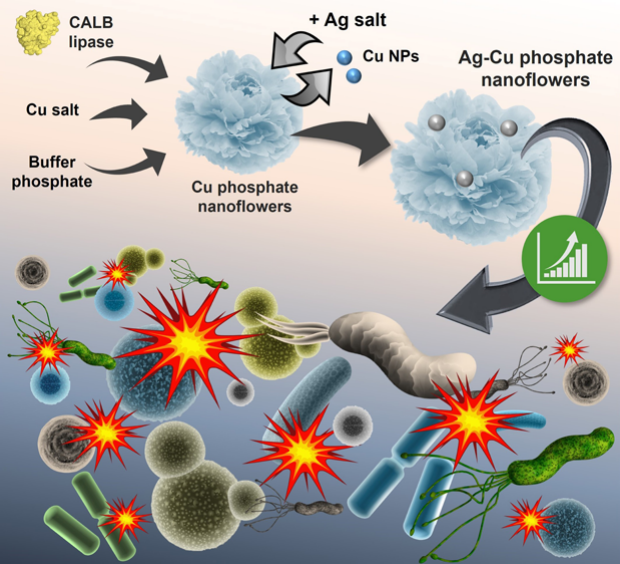

Infections caused by bacteria that are resistant to many drugs are a major threat to public health in many countries around the world. Here we demonstrate the creation of heterogeneous catalytic nanomaterials with outstanding antimicrobial properties against several superbugs. We have shown that replacing a small amount of copper in a generated copper-phosphate-enzyme nanoflower hybrid with silver drastically increases the antimicrobial capacity of the nanomaterial. In this sense, it has been confirmed that the exchange generated silver phosphate nanoparticles on the Cu nanoflowers, with control of the nanoparticle diameter size. The Fenton catalytic activity of the Ag-containing nanobiohybrids was affected, showing better performance with lower amounts of silver in the final hybrid. This effect was confirmed by their antimicrobial efficacy against *Escherichia coli*, where the Ag_4_Cu_32_@CALB hybrid displayed a log reduction of 3.9, an efficiency more than 5000 times higher than that obtained with copper nanoflowers (Cu_36_@CALB). The hybrid also showed excellent efficacy against other bacteria such as *Klebsiella pneumoniae*, *Pseudomonas aeruginosa*, and *Mycobacterium smegmatis* with log reductions of 7.6, 4.3, and 1.8, respectively.

## 1 Introduction

Antimicrobial resistance has recently emerged as a major global health concern. Recent studies have indicated that superbugs are responsible for over 4.95 million deaths annually.^1,2^ As bacteria evolve and develop resistance to traditional antibiotics, it is imperative that novel and innovative approaches be investigated to effectively address this growing crisis. A promising avenue for combating antimicrobial resistance is the utilization of nanomaterials.^3^ Characterized by their unique physical and chemical properties at the nanoscale, these materials exhibit properties such as an enhanced surface area, optimized drug delivery, and distinctive interactions with bacterial cells, which may prove beneficial in this regard. They have shown great potential in enhancing the effectiveness of antibacterial treatments^3^ and when applied as coating agent for avoiding transmission.^4,5^

Metal nanoparticles have been identified as a promising category of antibacterial agents due to their distinctive properties and multifaceted applications.^6−16^ A number of metals, such as silver and copper, have demonstrated remarkable antibacterial efficacy, thereby creating new avenues in the fight against microbial infections.^6−10^ Moreover, the use of combinations of different metals in the synthesis of nanoparticles offers a number of advantages over nanoparticles comprising a single metal.^11−14^ These advantages include the enhancement of properties, which gives rise to synergistic effects and tailored features; multifunctionality; stability; cost-effectiveness; and potential for novel applications. Indeed, bimetallic nanoparticles have been demonstrated to inhibit bacterial growth by employing a number of mechanisms, including (i) adhesion to the cell membrane, resulting in alterations to its structure, permeability, and deficiencies in cell functions such as ATP secretion and transport activity; (ii) entry into the cell and nucleus by disrupting mitochondrial function, destabilizing and denaturing proteins, ribosomes, and DNA interaction; and (iii) oxidase-like activity through the generation of reactive oxygen species (ROS), which can oxidize proteins, lipids, and DNA bases.^13,14^

In particular, copper and silver have been described as very efficient metals for combating microbial infections, with better performance than others, such as zinc. However, although silver is more effective, it also has higher toxicity and harmful properties.^7^ In fact, the majority of commercial materials are derived from the direct use or combination of copper or silver species, especially oxides at the bulk and micromolecular level. However, this approach has disadvantages in terms of the cost or efficiency.

Furthermore, the transfer of bacteria through contact with contaminated surfaces is an important pathway for the spread of infections. This has demonstrated the necessity to develop novel materials with antimicrobial activities to prevent the infection.^17^

Consequently, one option will be to develop new nanostructured materials combining both metals and controlling the percentage of one versus the other. The addition of silver as a secondary metal was considered suitable for this purpose, thanks to its recognized antibacterial properties.^18^ Furthermore, the combination of two metals could produce synergistic effects that increase the activity of bionanohybrids.^9,11,14^

Nevertheless, the majority of applications require the use of significant quantities of silver in the form of nanoparticles. Also, their instability tends to result in unfavorable agglomeration and/or decomposition.

One of the primary challenges is to develop bimetallic systems in which the silver content is minimal while maintaining a high efficiency. To achieve this, it is necessary to employ mechanisms that allow for the control and reduction of particle sizes to a minimum, with an optimal dispersion, high stability, and the prevention of aggregation. This approach allows for the exposure of the highest number of active sites on the surface of the nanoparticles, thereby enhancing the efficiency of the process.

In this context, the utilization of a biological molecule, as an enzyme-induced technology, to synthesize metal nanoparticles in a protein matrix at mild conditions can be regarded as an optimal methodology.^19−24^

The advantages described previously in terms of producing a highly catalytic efficient bimetallic systems can be possible through this technology in comparison with other systems where bimetallic nanoparticles are created in solutions or even supported on different materials.^11,12,14^ This is particularly important in processes such as the Fenton reaction, which generates reactive oxygen species (ROS), one of the ways to kill bacteria.

For example, the synthesis of highly stable and reusable copper nanoflowers was developed using a lipase as a matrix enzyme,^25,26^ which plays a special role in controlling the size and morphology of the generated metal nanoflower.^19−24^

Copper nanoflower systems have been shown to be highly effective against viruses.^25,26^ This type of material, which is excellent as a coating to prevent pathogens and infection, could therefore also be of interest for the problem of superbugs. In this way, the efficiency of the copper-only system against bacteria was first evaluated but with very poor results. The nanoflower system that allows AgNPs to be obtained on the surface with very small size and high degree of surface reactive active sites could represent an improved alternative.

**Figure 1:**
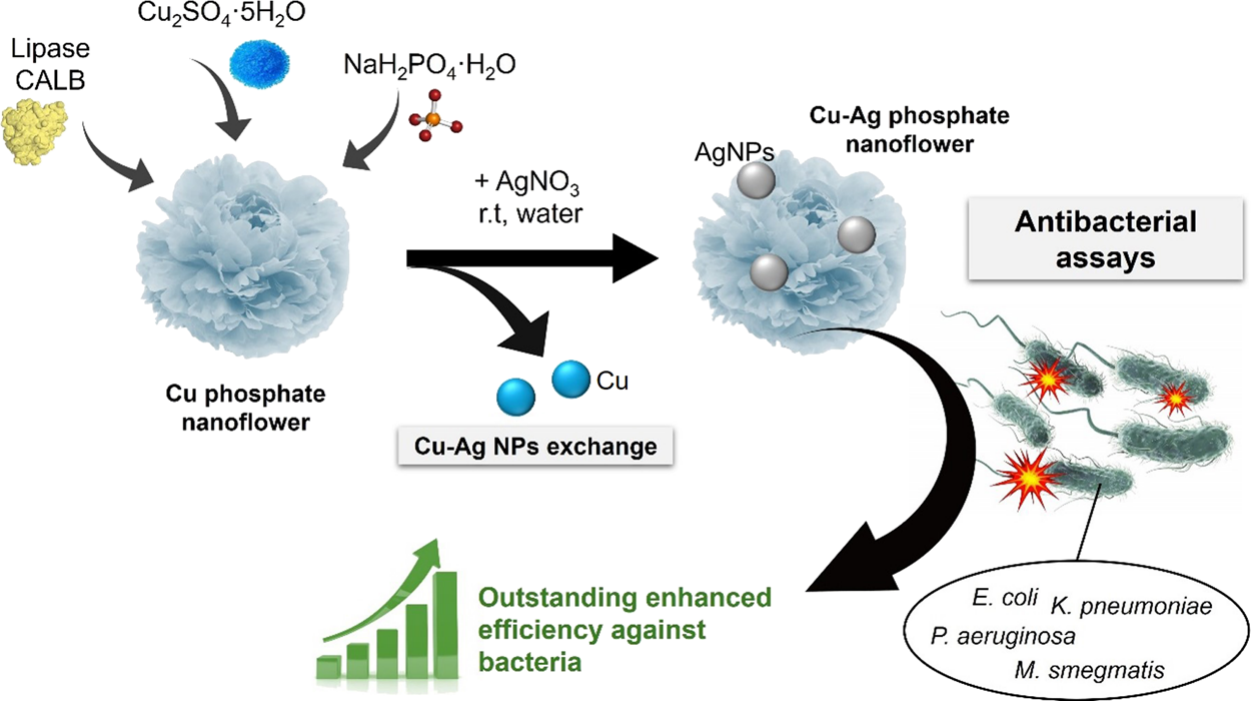
Scheme of Cu–Ag bionanohybrid synthesis and antimicrobial efficacy.

Therefore, in this work, we propose a new alternative to create a sustainable and stable bimetallic nanoparticle system, focused on the formation on stable Ag nanoparticles on the surface of a Cu phosphate-nanoflower macrostructure (Figure 1).

To evaluate their antibacterial efficacy, the new Cu–Ag bionanohybrids were exposed to several bacterial strains, including *Escherichia coli*, *Klebsiella pneumoniae*, and *Pseudomonas aeruginosa* (Figure 1). The strains were selected based on their designation in the literature; they are among the top six pathogens responsible for most deaths due to antimicrobial resistance.^2^ They were also tested against Gram-positive bacterium *Mycobacterium smegmatis*.

## 2 Experimental Section

### 2.1 Materials

The following materials were provided by Panreac (Barcelona, Spain): copper(II) sulfate pentahydrate (Cu_2_SO_4_·5H_2_O), hydrogen peroxide (H_2_O_2_, 33% v/v), sodium hydroxide (NaOH), and sodium dihydrogen phosphate 1-hydrate. The lipase B from *C. antarctica* (CAL-B) solution (Lipozyme CalB) was obtained from Novozymes (Copenhagen, Denmark). The following materials were purchased from Sigma-Aldrich: silver nitrate, *p*-nitrophenol, *p*-aminophenol, and sodium borohydride. The bacterial models utilized in this study were *Escherichia coli* C IJ1862 obtained from Prof. James J. Bull, *Klebsiella pneumoniae* from the Serum Statens Institute Collection, and *Pseudomonas aeruginosa* and *Mycobacterium smegmatis* mc2155 obtained from the ATCC collection.

### 2.2 Instrumentation

X-ray diffraction (XRD) patterns were obtained using a PANalytical X’Pert Pro polycrystalline X-ray diffractometer with a D8 Advance texture analysis Θ–Θ configuration (Bruker, Billerica, MA) with Cu Kα radiation (λ = 1.5406 Å, 45 kV, 40 mA). The X’Pert Data Viewer and X’Pert Highscore Plus were used for analysis.

X-ray photoelectron spectroscopy (XPS) spectra were obtained using an electronic spectroscopy system from SPECS GmbH with an ultrahigh vacuum (UHV) system (pressure about 10–10 mbar), a PHOIBOS 150 9MCD energy analyzer, and monochromatic X-ray sources. The analysis was performed with the CasaXPS program.

Transmission electron microscopy (TEM) studies were carried out using an S/TEM Titan 80-300 microscope equipped with a Cetcor Cs probe corrector and an energy dispersive X-ray spectrometer (EDS) for chemical composition analysis. Samples for TEM observation were prepared by dispersing a small amount of the material in ethanol, placing a drop of the suspension on a microscope nickel grid covered with a carbon film, and allowing the alcohol to evaporate. The samples were then dried and cleaned with a plasma cleaner. TEM (bright-field BF, dark-field DF, and selected area diffraction) and STEM modes (BF detector to show structure and morphology; high-angle dark-field (HAADF) detector to show chemical contrast (*Z*-contrast)) were used for imaging. As the tested material was sensitive to the electron beam, the intensity of the electron beam was measured during microscopic observations, and the exposure time were limited.

Inductively coupled plasma atomic emission spectrometry (ICP) was performed on the solid material using an AVIO 220MAX equipment (PerkinElmer, Waltham, MA). Five milligrams of the solid powder was treated with 5 mL of HCl (37% v/v) for digestion. Then, it was added with 5 mL of water and centrifuged, and the clear solution was analyzed for Cu and Ag content. ICP-OES analyses were performed by quintupled.

A Biocen 22 R (Orto-Alresa, Ajalvir, Spain) refrigerated centrifuge and a Basic Research Telstar LyoQuest freeze-dryer (Steinfurt, Germany) were used in the synthesis of the hybrids. Spectrophotometric analyses were run on a V-730 spectrophotometer (JASCO, Tokyo, Japan). Chromatographic analyses were run by using an HPLC system equipped with a pump (PU-4180, JASCO, Tokyo, Japan) and a UV-4075 UV–vis detector (JASCO, Tokyo, Japan).

### 2.3 General Synthesis of Ag(I)-Cu(II)-Enzyme NanoFlower hybrids

A 180 mg portion of commercial Lipase B from *Candida antarctica* (CAL-B) solution was added to 600 mL of sodium phosphate buffer (0.1 M, pH 7) in a 1 L glass bottle containing a small magnetic bar stirrer at 345 rpm. Then, 6 g of Cu_2_SO_4_·5H_2_O (10 mg mL^–1^) was added to the solution, and the mixture was incubated for 1 h at room temperature. After this time, a light blue emulsion with a concentration of 5000 ppm of the copper bionanohybrid was obtained.^19^ The mixture was then centrifuged at 8000 rpm for 8 min; the supernatant was removed, and the generated pellet was recollected and resuspended in the same initial volume with distilled water (600 mL, 5000 ppm). This hybrid is the so-called Cu_36_@CALB.

Then, to generate the silver–copper bionanohybrids, different amounts of silver nitrate (120, 60, 12, 7.2, or 4.8 mg) were directly added to 20 mL of this Cu_36_@CALB) solution (pH 6.5). In each case, the mixture was stirred at 345 rpm for 24 h at room temperature. After that, the mixture was centrifuged at 8000 rpm for 8 min at 20 °C. Then, the supernatant was removed, and the pellet was resuspended in 15 mL of distilled water and centrifugated again. This process was repeated two more times more. Finally, the recovered pellet was resuspended in 2 mL of distilled water, collected in a cryotube, frozen with liquid nitrogen, and lyophilized at −52 °C and 0.01 mbar for 16 h. Eight different bionanohybrids were obtained as a solid powder, and they were named according to the copper and silver content (% wt) obtained by ICP-OES: Ag_15_Cu_21_@CALB, Ag_8_Cu_28_@CALB, Ag_4_Cu_32_@CALB, Ag_2_Cu_34_@CALB, and Ag_1.5_Cu_35_@CALB. The color of the hybrids lightened from light blue to gray as the amount of silver nitrate increased.

### 2.4 Activity assay of Hybrids in the Fenton Oxidation of the *p*-Aminophenol Reaction

*p*-Aminophenol (pAP) (1 mg) was dissolved in 10 mL of distilled water, and 100 mM of hydrogen peroxide (1%, v/v) was added at r.t. To initialize the reaction, 3 mg of the hybrid was added to the solution, and the mixture was stirred gently at room temperature on an orbital shaker (320 rpm). At different times, samples (100 μL) were taken, and the reaction was followed by HPLC analysis. Samples were first centrifuged at 8000 rpm for 5 min, and then 50 μL of liquid was diluted 10 times in the mobile phase before injection. The HPLC column was a C8 Kromasil 150 × 4.6 mm AV-2059. HPLC conditions were as follows: an isocratic mixture of 10% (v/v) acetonitrile and 90% bidistilled water at pH 4.0 as mobile phase, UV detection at 254 nm, and a flow rate of 0.7 mL/min. Under these conditions, retention times of H_2_O_2_ and pAP were 3.0 and 3.8 min, respectively. The possible adsorption of the substrate on the catalyst was first tested, and without the presence of hydrogen peroxide, no reaction was observed, and the full area of the substrate was unchanged in the HPLC analysis.

### 2.5 Antibacterial Assays

The bacterial viability in the presence of the bionanohybrid was tested against different bacterial models. The bacteria used for this experiment were *Escherichia coli* C IJ1862, *Klebsiella pneumoniae,Pseudomonas aeruginosa*, and *Mycobacterium smegmatis*.

First, 2.5 or 8 mg of each nanohybrid was added to 2 mL of Milli-Q water, and the mixture was stirred well, getting a final concentration of 1250 or 4000 ppm, respectively. Then, 50 μL of the well-stirred bionanohybrids suspensions was added to 450 or 1000 μL of the LB broth and a stationary bacterial culture mixture (obtaining final concentrations of 125 and 200 ppm for the bionanohybrids). One milligram of the hybrids was added directly to 1 mL of LB broth and a stationary bacterial culture mixture to obtain the final concentration of 1000 ppm.

The mixtures were thoroughly vortexed and incubated for 4 h at 25 °C and 800 rpm. Each sample and bacterial condition were tested in triplicate. From each triplicate, serial dilutions were performed to facilitate the subsequent counting of isolated colonies. After 4 h of incubation, 100 μL of each sample was added to an LB + CaCl_2_ Petri dish with glass beads. Plates were incubated at 37 °C for 24 h (*E. coli*, *P. aeruginosa*, and *K. pneumoniae*) and 72 h (*M. smegmatis*), and then isolated colonies were counted. The concentration (CFU/mL) of each replicate was calculated for each condition following eq 1 and considering the colony count at the optimal dilution and the plated volume. After that, the weighted average was calculated. A negative control (LB) and a positive control (LB with bacteria) were done in parallel in the absence of the bionanohybrid to determine the bacterial death over time. No cell toxicity of the bionanohybrids was observed.
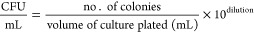
1

## 3 Results and Discussion

### 3.1 Synthesis and Characterization of Ag/Cu-Enzyme Hybrids

The silver-copper-CALB hybrids were synthesized by adding silver nitrate to an aqueous solution of the previously synthesized bionanohybrid of Cu (Cu_36_@CALB hybrid) (see the Supporting Information).^19^

In this hybrid, the copper content is 36% (w/w) as measured by ICP-OES. Different amounts of silver salt (6, 3, 0.6, 0.36, and 0.24 mg per mL) were added to an aqueous solution of this heterogeneous Cu hybrid (5000 ppm concentration), and the reaction was incubated at room temperature for 24 h. The selection of the amounts of silver was made according to data from our previous research, where the concentration of silver required to generate an Ag-CALB hybrid was defined as 50 mg of Ag salt per mg of enzyme.^27^

In any case, no synthesis was observed using the previous amounts of silver with the CALB enzyme instead of the Cu enzyme nanoflower (data not shown).

Five different Ag-containing Cu hybrids were obtained, and the silver and copper contents were determined by ICP-OES (Table 1). The new hybrids were named according to the amount of metal, i.e., Ag_15_Cu_21_@CALB, Ag_8_Cu_28_@CALB, Ag_4_Cu_32_@CALB, Ag_2_Cu_34_@CALB, and Ag_1.5_Cu_35_@CALB (Table 1).

**Table 1:** Ag and Cu Contents of the Different Bionanohybrids

bionanohybrid	Ag added to Cu_36_@CALB (wt %)	Ag amount^a^	Ag (wt %)^b^	Cu (wt %)^b^
Ag_15_Cu_21_@CALB	50%	6	15.0	21.3
Ag_8_Cu_28_@CALB	25%	3	8.1	28.3
Ag_4_Cu_32_@CALB	5%	0.6	3.7	32.3
Ag_2_Cu_34_@CALB	3%	0.36	2.0	34.1
Ag_1.5_Cu_35_@CALB	2%	0.24	1.5	34.7

a Amount in milligrams of silver nitrate added to each milliliter of Cu_36_@CALB hybrid solution (5 g/L).

b Metal content in each milligram of solid determined by ICP-OES analysis.

According to the ICP results, there seems to be a clear exchange of Cu ions in the Cu_36_@CALB hybrid, which are replaced by Ag ions. This exchange was proportional, maintaining the final metal content in the enzyme-metal nanoflower (Table 1). This finding is consistent with previous research on copper metalloproteins, which suggests that Ag^+^ may compete with endogenous copper when the active site is positioned in a solvent-exposed medium. This process involves the release of Cu^2+^ into the surrounding medium, resulting in an Ag → Cu substitution.^28,29^

**Figure 2:**
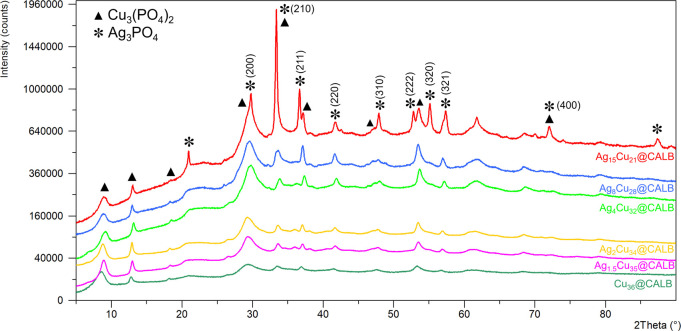
Powder X-ray diffraction (XRD) patterns of different Ag–Cu bionanohybrids.

To characterize the various new hybrids, powder X-ray diffraction (XRD) analysis was first performed (Figure 2). The XRD patterns of the hybrids with lower Ag content (Ag_4_Cu_32_@CALB, Ag_2_Cu_34_@CALB, and Ag_1.5_Cu_35_@CALB) showed profiles similar to those of the initial copper nanoflower one (copper phosphate pattern, Figure S1) but with small diffraction peaks corresponding to silver (i) phosphate, particularly at 32 and 36° (Figure 2), which agree well with the peaks attributed to the body centered cubic structure of silver phosphate (JCPDS, card no. 6-505).

These peaks were indicative of the presence of silver phosphate in the sample, which was observed much more clearly as the amount of silver in the sample increased (25 and 50%, Table 1). In the XRD pattern, the crystalline diffraction peaks at 29° (110) and 48° are the main peaks for Cu phosphate, whereas the peaks at 32 and 36° or the representative trident of diffraction peaks in silver phosphate, 52.5, 55, and 57.5°, was well-defined (Figure 2).

X-ray photoelectron spectroscopy (XPS) analysis confirmed the presence of Ag(I) and Cu(II) as metal species (Figure S2).

**Figure 3:**
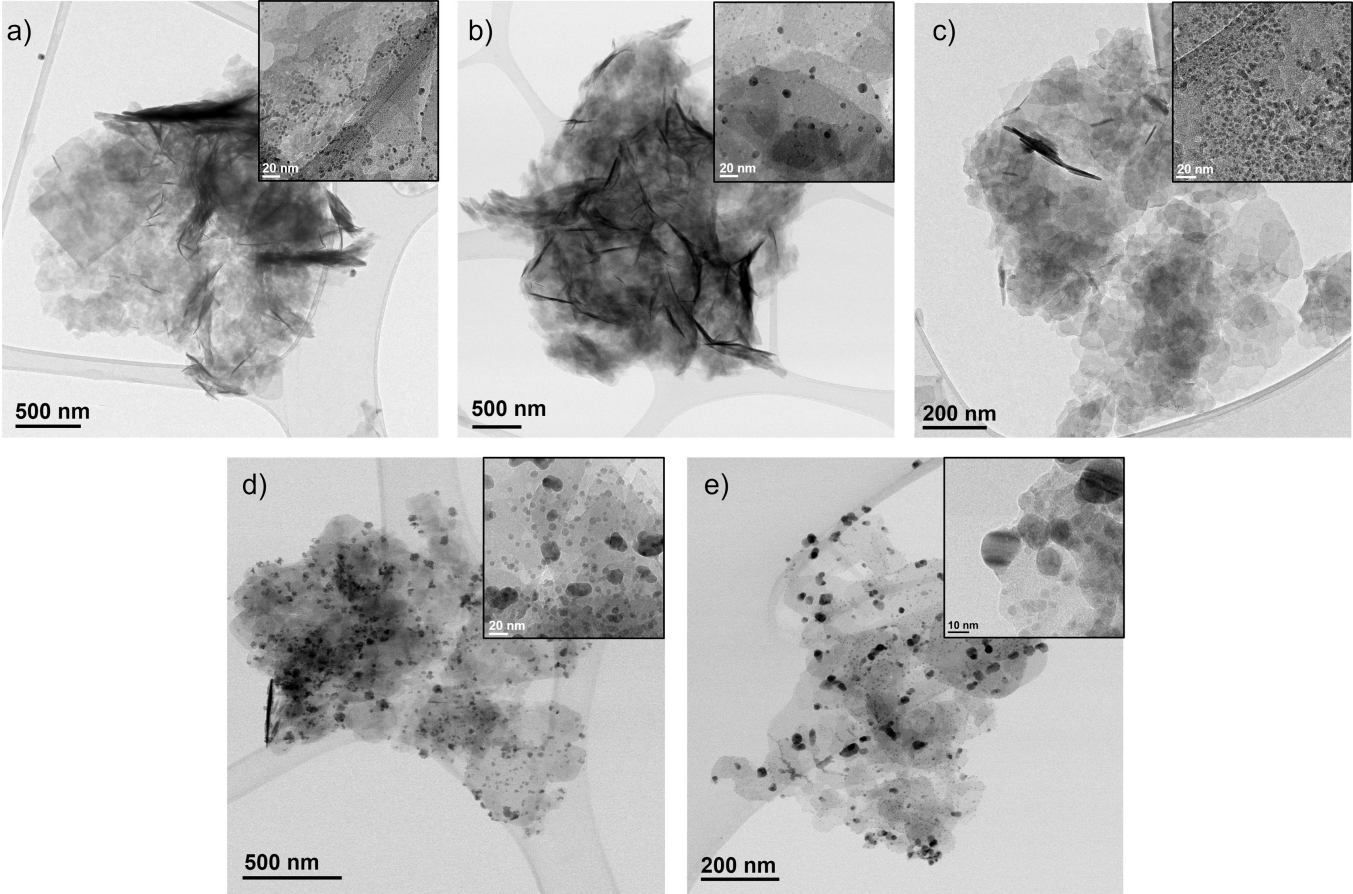
Transmission electron microscopy (TEM) images of Ag–Cu bionanohybrids captured at different resolutions. Inset images taken at 10 or 20 nm clearly depict NP size. (A) Ag_1.5_Cu_35_@CALB. (B) Ag_2_Cu_34_@CALB. (C) Ag_4_Cu_32_@CALB. (D) Ag_8_Cu_28_@CALB. (E) Ag_15_Cu_21_@CALB.

TEM analyses were performed to characterize the nanostructures in the different hybrids (Figure 3, Figures S3–S7). In terms of supramolecular structure, the nanoflowers were well-defined in the Ag/Cu hybrids with lower silver content (Ag_1.5_Cu_35_@CALB, Ag_2_Cu_34_@CALB, and Ag_4_Cu_32_@CALB) (Figure 3a–c), whereas the formation of nanosponges seems to be obtained after the higher amount of silver substitution (Ag_8_Cu_28_@CALB, Ag_15_Cu_21_@CALB) (Figure 3d,e).

The formation of larger metal nanoparticles up to the well-formed Cu phosphate nanoparticles of around 4 nm (Figure S1) was observed in all cases. However, the average diameters of these nanoparticles (presumably silver) became larger and larger as the amount of silver was increased (Figure 3). In the case of Ag_1.5_Cu_35_@CALB, nanoparticles with a diameter of around 7 nm were formed (Figure S3), whereas in the case of Ag_2_Cu_34_@CALB and Ag_4_Cu_32_@CALB, nanoparticles of around 9 nm were obtained (Figure 3b,c, Figures S4 and S5). In the case of Ag_8_Cu_28_@CALB, Figure 3d shows nanoparticles with an average diameter size of 14 nm (Figure S6). The largest nanoparticles were observed in the case of the Ag_15_Cu_21_@CALB hybrid with an average diameter size of 16 nm (Figure 3e, Figure S7).

**Figure 4:**
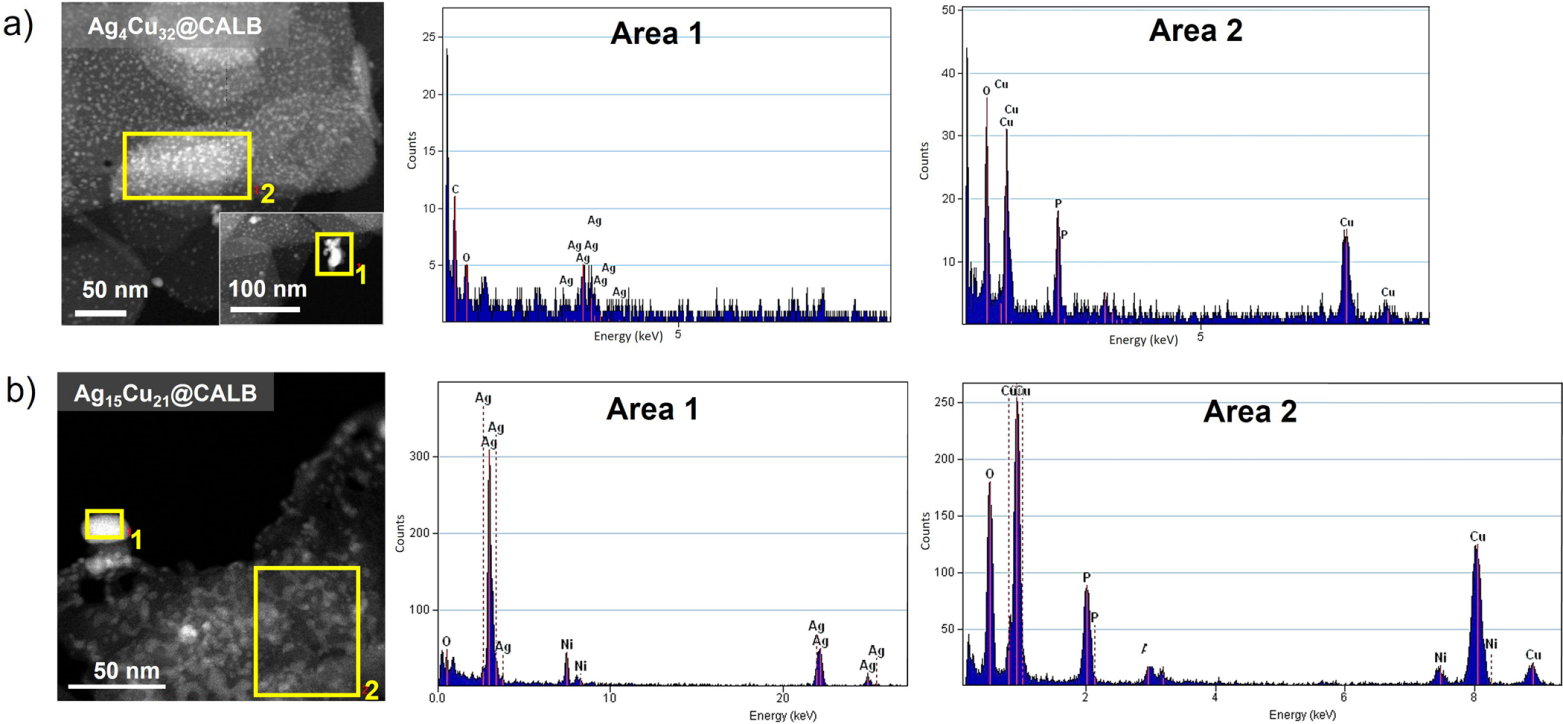
HAADF STEM imaging (left panels) and STEM-EDX analysis (right panels) of Cu and Ag nanoparticles. (A) Ag_4_Cu_32_@CALB hybrid. (B) Ag_15_Cu_21_@CALB hybrid. Area code of STEM-EDX: Ag particles in Area 1, Cu particles in Area 2. The nickel signal corresponds to the grid used for the experiment.

High-angle annular dark-field scanning transmission electron microscopy (HAADF-STEM) analysis was carried out, confirming that these larger nanoparticles actually corresponded to silver phosphate, whereas the smaller ones corresponded to copper phosphate (from the nanoflower). Furthermore, this method also revealed that the macrostructure is composed of both nanoparticles, with the copper ones embedded in the nanoflower and the silver ones on the surface of the flower (Figure 4, Figure S8).

### 3.2 Fenton Catalyst

**Figure 5:**
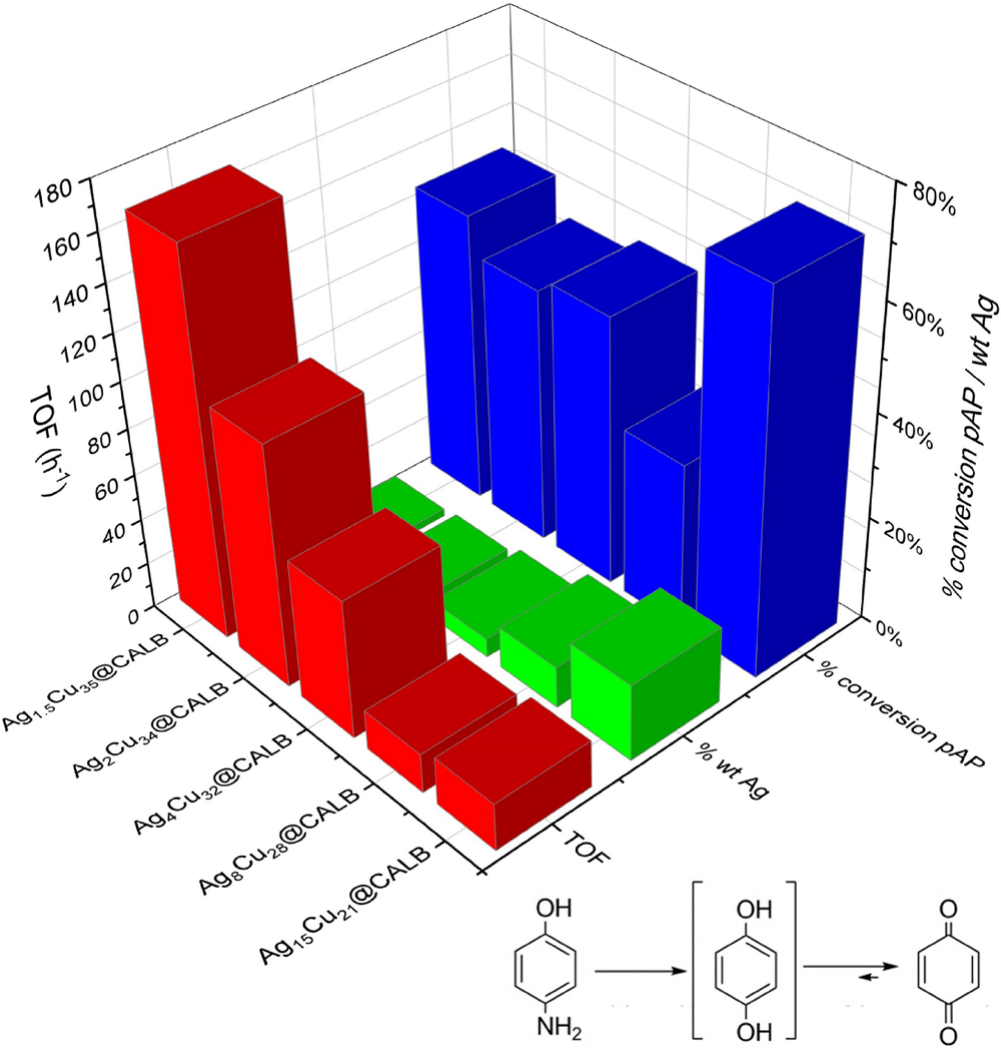
Fenton catalysis by different nanobiohybrids in the hydroxylation reaction of *p*-aminophenol to benzoquinone. Conversion of different Cu hybrids after a 5 min reaction. Conditions: 100 mg/L of pAP in 10 mL of distilled water, 1% v/v of H_2_O_2_, and 3 mg of hybrid at room temperature.

The selective hydroxylation of *p*-aminophenol to benzoquinone using hydrogen peroxide as the green oxidant was used (Figure 5, Figure S9).

This reaction represents a mimetic indirect assay of the ROS capacity of the nanozymes (Fenton-type reaction), which has recently been successfully used to confirm the antimicrobial and antiviral activity of various nanozymes.^19,20^

The Fenton process was observed for all of these Ag/Cu hybrids. In most cases, more than 50% conversion was obtained in 5 min (Figure 5), with 90% conversion after 10 min (Figure S9). However, if the comparison is made by the amount of Ag in the hybrids and by the turnover frequency (TOF) value, and it is clear that reducing the amount of silver seems to be positive. This could be due to the presence of smaller silver nanoparticles with a larger surface area and therefore the highest efficiency. Both parameters need to be considered for the final efficiency.

### 3.3 Antibacterial Assays

**Figure 6:**
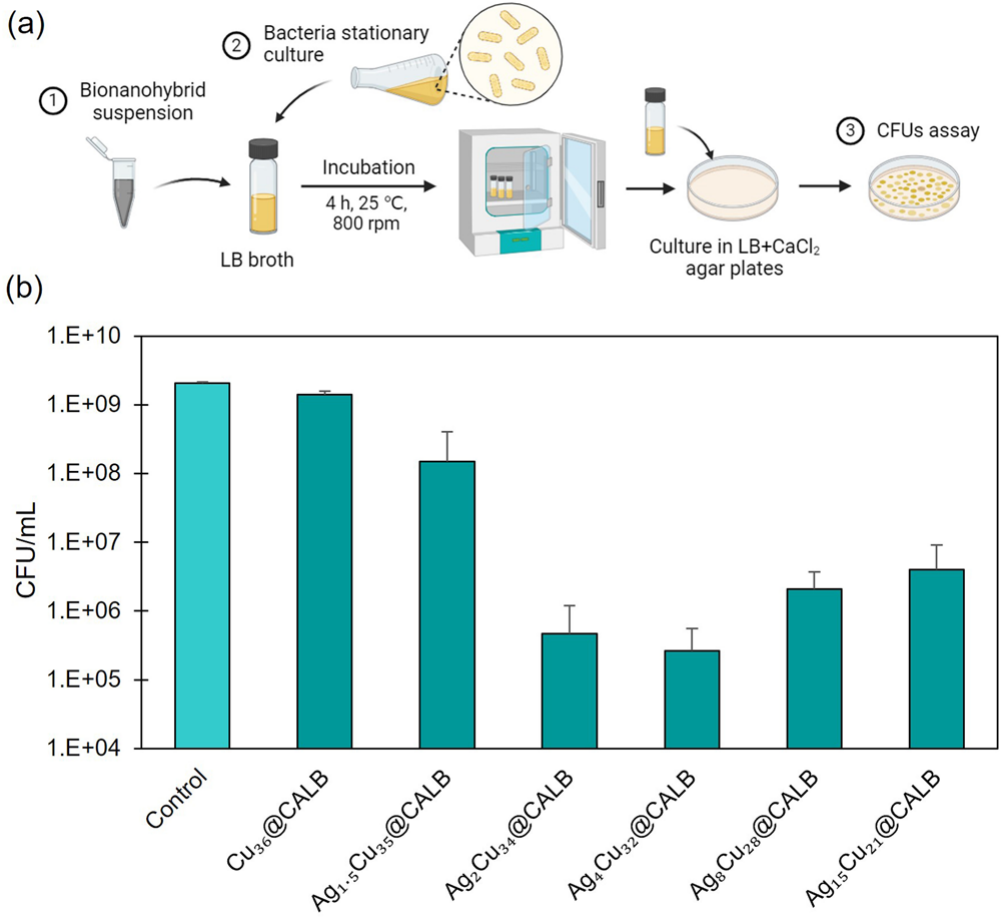
(a) Illustrative scheme of the method. Created with BioRender.com. (b) Reduction in bacterial concentration (measured viable bacteria as CFU count per mL in the presence of the bionanohybrid relative to the control in the absence of the bionanohybrid) after a 4 h incubation with a concentration of 125 ppm of each hybrid against *E. coli*. The graph shows the average of three different assays in CFU/mL. Error bars correspond to standard deviation.

Antimicrobial analyses were conducted using an *E. coli* strain, with colony-forming units (CFU) enumerated following the incubation of a stationary bacterial culture for 4 h with each bionanohybrid at a concentration of 125 ppm (see Figure 6). This methodology is based on previously developed procedures.^30^

Cu_36_@CALB was tested, and a slight inhibition was observed. The incorporation of silver in the new hybrids greatly increased the antimicrobial efficacy of this Cu hybrid, almost 10 times for Ag_1.5_Cu_35_@CALB, and extremely high improvements were observed for Ag_2_Cu_34_@CALB and Ag_4_Cu_32_@CALB, being 3000 and >5000 times more effective than Cu_36_@CALB, respectively (Figure 6). Higher amounts of silver also showed good improvement, i.e., almost 700 times for Ag_8_Cu_28_@CALB hybrid and 300 times for Ag_15_Cu_21_@CALB. The latter results show how increasing the amount of silver (>20%) leads to lower efficiencies than smaller amounts of silver; the higher the amount is, the lower is the efficiency. In fact, it seems to indicate that the presence of 4% silver in the system corresponds to the most suitable options.

Thus, the Ag_4_Cu_32_@CALB hybrid achieved a bacterial reduction of 3.9 log_10_ (Figure 6).

In contrast, a number of studies have demonstrated that the size of silver nanoparticles (Ag NPs) is not the sole determinant of their antibacterial efficacy. Other critical factors, including the release of ions, surface charge, and the capacity to generate reactive oxygen species (ROS), also influence this effect.^31−33^

**Figure 7:**
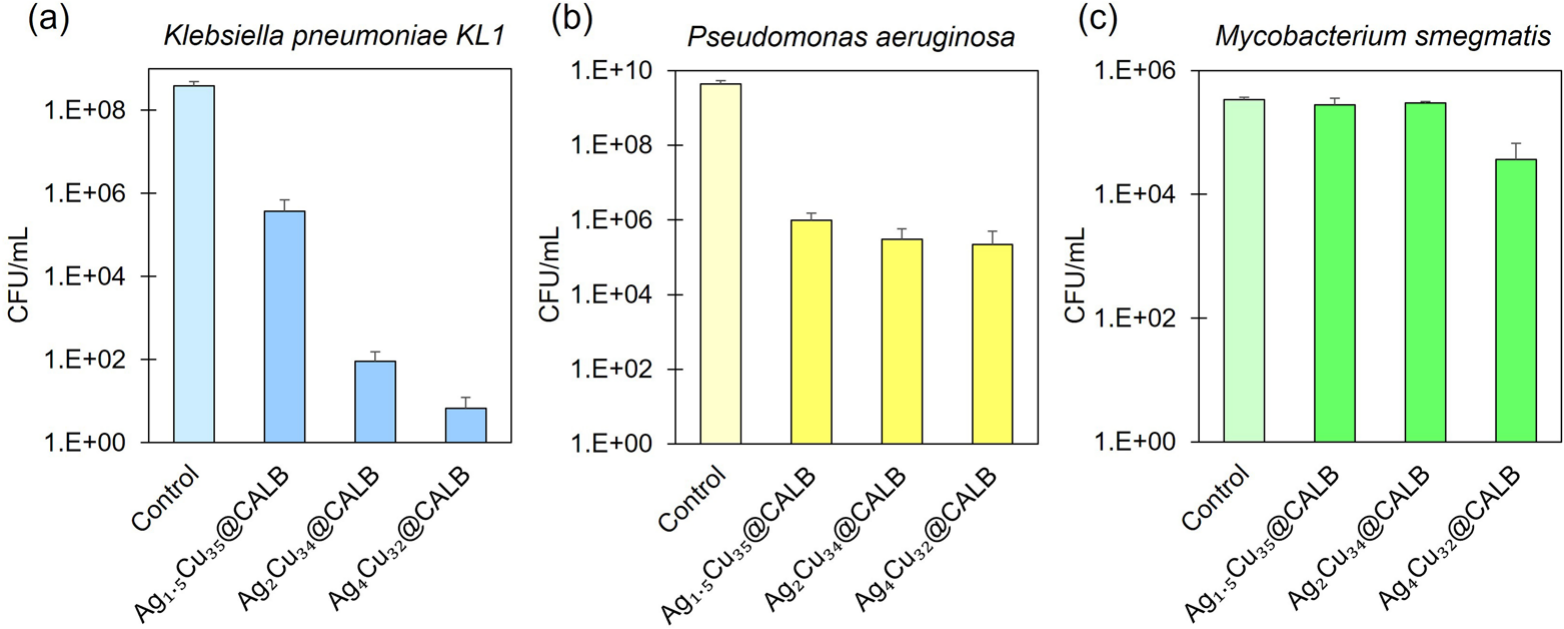
Reduction in bacterial concentration (measured viable bacteria as CFU count per mL in the presence of the Ag–Cu bionanohybrid relative to the control in the absence of the bionanohybrid) after a 4 h incubation with a concentration 1000 ppm of each hybrid. (a) *Klebsiella pneumoniae*, (b) *Pseudomonas aeruginosa*, and (c) *Mycobacterium smegmatis*. The graph shows the average of three different assays in CFU/mL. Error bars correspond to standard deviation.

To further examine this hypothesis, Ag/Cu bionanohybrids with lower silver content (Ag_4_Cu_32_@CALB, Ag_2_Cu_34_@CALB, and Ag_1.5_Cu_35_@CALB) were selected for additional experiments with *Pseudomonas aeruginosa* and *Klebsiella pneumoniae*, two Gram-negative bacteria currently of public health concern, and a Gram-positive mycobacterium, *Mycobacterium smegmatis* (Figure 7). The different strains were incubated with the hybrids for 4 h at a hybrid concentration of 1000 ppm.

For *K. pneumoniae*, the inhibition profile was similar to that of *E. coli*, with biocidal capacity for all the hybrids tested but with the highest inhibition value observed for Ag_4_Cu_32_@CALB with 7.8 log_10_ reduction (Figure 7a). Ag_2_Cu_34_@CALB also showed a high efficiency, 6.6 log_10_ reduction, whereas Ag_1.5_Cu_35_@CALB showed a reduction of 2 log_10_. In the case of *P. aeruginosa*, the differences between Ag_4_Cu_32_@CALB, Ag_2_Cu_34_@CALB, and Ag_1.5_Cu_35_@CALB were less significant, reaching high log reduction values in all cases, approaching to 4 log_10_ bacterial reduction (Figure 7b).

**Figure 8:**
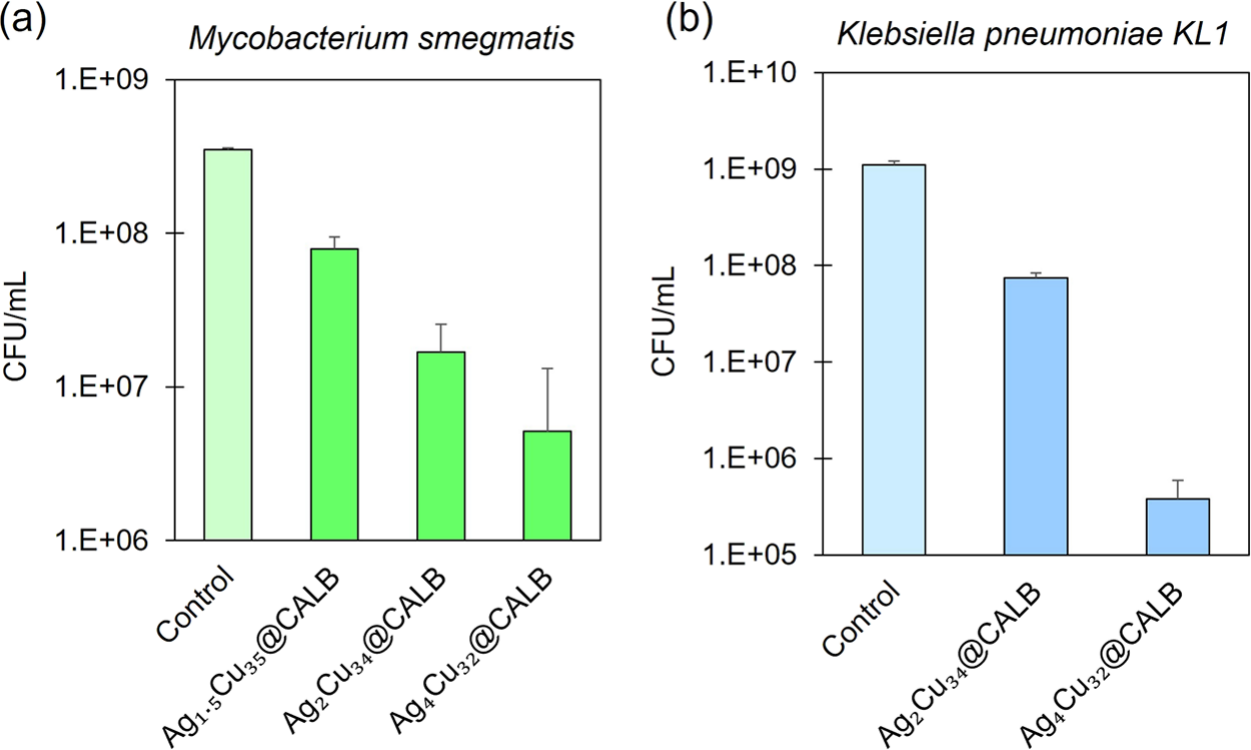
Reduction in bacterial concentration (measured as CFU count per milliliter) in the presence of the Ag–Cu bionanohybrid relative to the control in the absence of the bionanohybrid. (A) After a 16 h incubation with a concentration of 1000 ppm of each hybrid against *M. smegmatis*. (B) After a 4 h incubation with a concentration 200 ppm of each hybrid against *K. pneumoniae*. The graph shows the average of three different assays in CFU/mL. Error bars correspond to the standard deviation.

The antibacterial effect against *M. smegmatis* was significantly lower compared with the other bacteria. Nevertheless, Ag_4_Cu_32_@CALB achieved 89.2% inhibition (Figure 7c). However, no significant antibacterial effect against *M. smegmatis* was observed with the hybrids Ag_2_Cu_34_@CALB, Ag_1.5_Cu_35_@CALB, and Ag_1_Cu_35_@CALB. This phenomenon can be attributed to the Gram-positive nature of *M. smegmatis*, which differentiates it from Gram-negative bacteria. The peptidoglycan layer of *M. smegmatis* renders it more resilient to bionanohybrid assault than Gram-negative bacteria, which exhibit a thinner peptidoglycan layer and an outer layer comprising lipopolysaccharides, lipoproteins, and lipids.^34^ To ascertain whether a longer exposure of the hybrids to *M. smegmatis* would yield a more pronounced effect, a second experiment was conducted for 16 h of incubation, maintaining the 1000 ppm hybrid concentration. The results of the experiment demonstrated an enhancement in the antibacterial efficacy of all the hybrids (Figure 8a).

The Ag_4_Cu_32_@CALB hybrid showed the highest activity, achieving almost a 2 Log reduction, whereas Ag_2_Cu_34_@CALB and Ag_1.5_Cu_35_@CALB achieved inhibition rates of 95.2 and 77.3% respectively.

On the other hand, it was tested whether reducing the concentration of the hybrids against *K. pneumoniae* had any effect on their efficacy. For this purpose, Ag_4_Cu_32_@CALB and Ag_2_Cu_34_@CALB were selected, being the best hybrids against *K. pneumoniae* in the 1000 ppm experiment. The concentration of the hybrids was set at 200 ppm, and the exposure time was set at 4 h (Figure 8b). Ag_4_Cu_32_@CALB also showed high antibacterial efficacy under these conditions, with 3.5 log_10_ bacterial reduction with higher differences compared to the results for Ag_2_Cu_34_@CALB, which showed a slight improvement in this case with 1.2 log_10_ reduction (Figure 8b).

The results indicate that the incorporation of substantial quantities of silver into the hybrids does not yield hybrids with enhanced antibacterial properties. This phenomenon is attributed to the formation of larger silver phosphate species, which impedes the optimal interaction with the bacterial membrane. Additionally, a notable reduction in the silver content of the novel hybrids (e.g., Ag_1.5_Cu_35_@CALB) results in a considerable decline in antibacterial efficacy.

Thus, the best and most efficient antibacterial material was the Ag_4_Cu_32_@CALB hybrid.

Therefore, in terms of the mechanism of action against bacteria, these results showed that the compromise between the lower amount of silver content and the final very small AgNPs located on the high surface area of the Cu nanoflowers produces a nanozyme with an extremely improved antimicrobial activity.

Therefore, the synergistic effect could be due to the efficiency of AgNPs to destabilize the cell wall structure, causing membrane damage through direct interaction with membrane proteins,^35−38^ which allows the generation of reactive oxygen species (ROS), mainly the intermediate ·OH radical species,^22^ to be enhanced by copper nanoparticles. The synergistic effect is also similar to that shown in other systems, where it has been demonstrated that, in the presence of Cu^2+^, there is less binding of Ag^+^ to the proteins of the incubation media.^39^

In terms of morphology, the high surface area of the nanoflowers is essential for the final activation of copper efficiency, whereas the NP surface is critical for the highest antimicrobial activity of silver. Therefore, the highest Ag content in the hybrid resulted in a loss of the nanoflower structure, and larger and more aggregated nanoparticles were formed on the nanoflower surface, which could be related to the lowest efficiency compared with the hybrid with 4% Ag content.

In fact, additional experiments were carried out with the highest amount of silver content to prepare a hybrid containing mainly silver, producing a hybrid with a 32% Ag content of Ag (+) phosphate species (Figure S9). Thus, this hybrid showed more than two times lower TOF values in the Fenton process than Ag_4_Cu_32_@CALB and therefore lower antimicrobial efficiency against *E. coli* bacteria (Figure S10), reinforcing previous results.

## 4 Conclusions

A simple and versatile synthesis method for stable bimetallic Ag(I)/Cu(II) phosphate bionanohybrid nanomaterials has been developed by generating exposed silver NP surfaces on a copper phosphate nanoflower hybrid. It was also found that the amount of silver added is a critical factor in the formation of the bionanohybrids.

The concentration of silver in the hybrids was found to have a significant impact on the nanoparticle size and aggregation state. Higher silver concentrations resulted in the formation of hybrids containing larger and more aggregated nanoparticles, which ultimately influenced the efficiency. In contrast, lower silver concentrations led to the production of hybrids comprising smaller, less aggregated, and more accessible silver nanoparticles, thereby enhancing their activities. This observation was corroborated by antibacterial assays against various superbugs. The presence of silver in the Ag_4_Cu_32_@CALB hybrid allowed one to obtain a nanomaterial with more than 5000 times more efficiency than the copper nanoflowers (Cu_36_@CALB), with a log bactericidal reduction of 3.9 against *Escherichia coli*. Extremely huge results of biocide activity were also obtained with this hybrid against other important superbugs, i.e., *Klebsiella pneumonia* and *Pseudomonas aeruginosa*, with the best log reductions of 7.8 and 4.3, respectively. Even for *M. smegmatis*, a Gram-positive bacterium, biocidal efficacy was observed for Ag_4_Cu_32_@CALB. Based on the results, it can be concluded that the bionanohybrid Ag_4_Cu_32_@CALB is an improvement over Cu_36_@CALB, as it significantly enhances its antibacterial activity. The addition of 0.6 mg of silver per milliliter of Cu_36_@CALB was shown to yield a highly effective antimicrobial material. These findings open up exciting possibilities for developing an antibacterial super material that could also kill other pathogens like viruses and fungi.

## Supporting Information Available

Figures of characterization of the hybrids (Figures S1–S8), profile of pAP oxidation reaction catalyzed by nanobiohybrids (Figure S9), characterization of the Ag_32_ hybrid (Figure S9), and catalytic and antimicrobial activity of the Ag_32_ hybrid (Figure S10) (PDF)
