# Supplementary material for: Low Silver/Copper Exchange in a Copper-Phosphate Enzyme Nanoflower Hybrid Extremely Enhanced Antimicrobial Efficacy against Multidrug Resistant Bacteria
Source: ACS Appl Bio Mater. 2024 Sep 10;7(10):6740–8. doi: 10.1021/acsabm.4c00898 (PMC11497203; doi:10.1021/acsabm.4c00898)
Supplement: Supplementary file 1 — mt4c00898_si_001.pdf [file mt4c00898_si_001.pdf]

Supporting Information

Low Silver/copper Exchange in Copper-phosphate Enzyme Nanoflower Hybrid Extremely Enhanced Antimicrobial Efficacy against Multi-drug Resistant Bacteria

Clara Ortega-Nieto,[1] Noelia Losada-Garcia,[1] Pilar Domingo-Calap,[2] Miroslawa Pawlyta[3] and Jose M. Palomo*[1]

[1]Instituto de Catálisis y Petroleoquímica (ICP). CSIC. 28049, Madrid, Spain

[2]Institute for Integrative Systems Biology (I[2]SysBio). Universitat de València-CSIC 46980, Paterna, Spain

[3]Materials Research Laboratory, Faculty of Mechanical Engineering, Silesian University of Technology, Konarskiego 18A, 44-100, Gliwice, Poland

*Correspondence: josempalomo@icp.csic.es

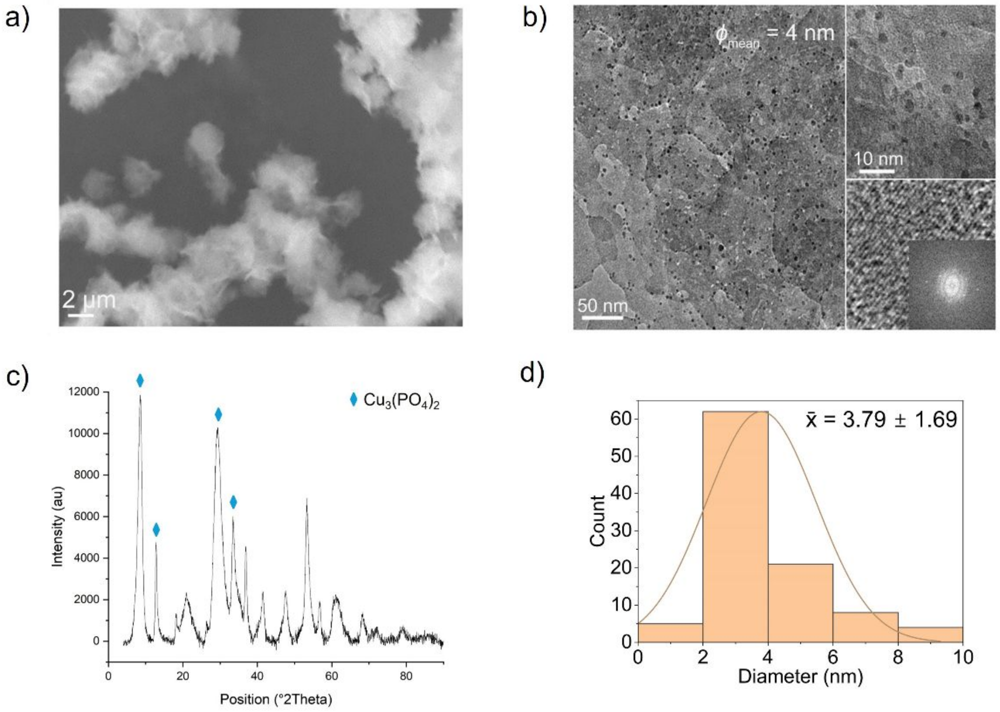

Figure S1. Characterization of Cu_{36}@CALB. A) SEM analysis. B) TEM images. C) XRD pattern. D) Particle size distribution.

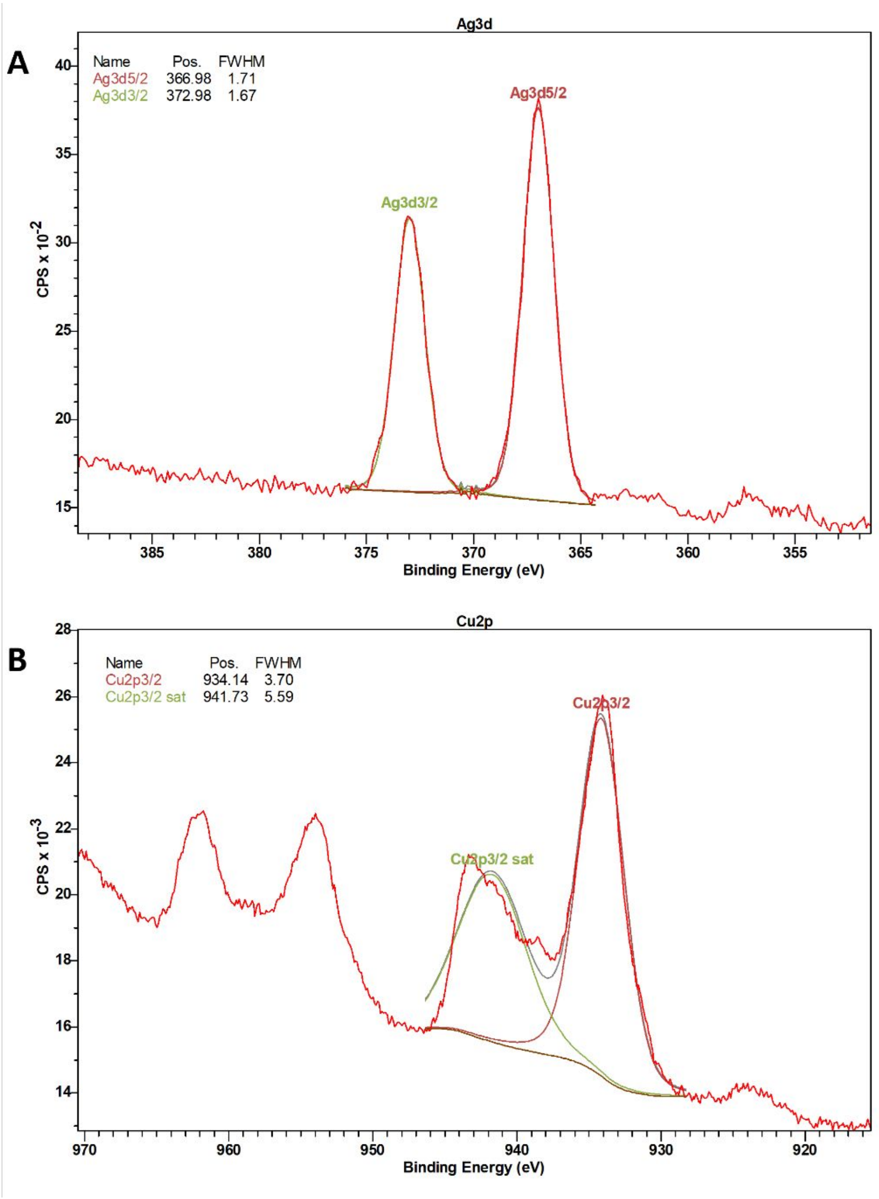

ç

Figure S2. XPS analysis of Ag_4Cu_{32}@CALB. A) Ag3d spectrum. B) Cu2p spectrum.

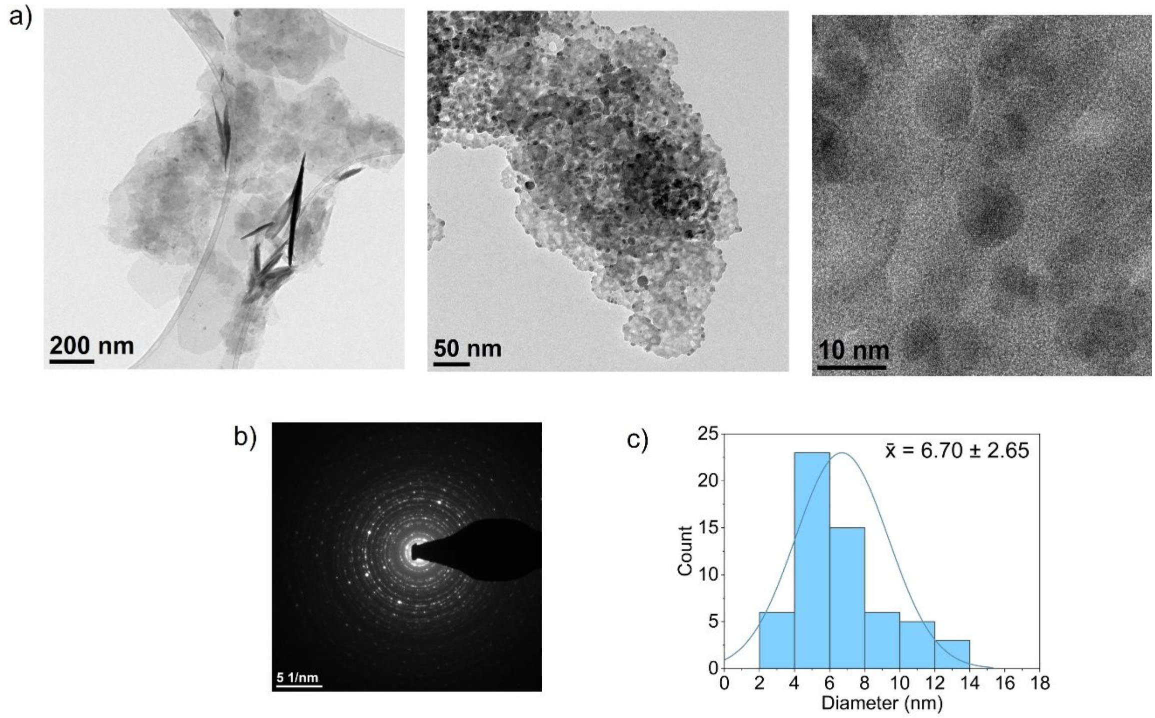

Figure S3. Characterization of $Ag_{1.5}Cu_{35}$@CALB bionanohybrid. A) TEM images. B) Fast Fourier transform (FFT) pattern.C) AgNPs size distribution.

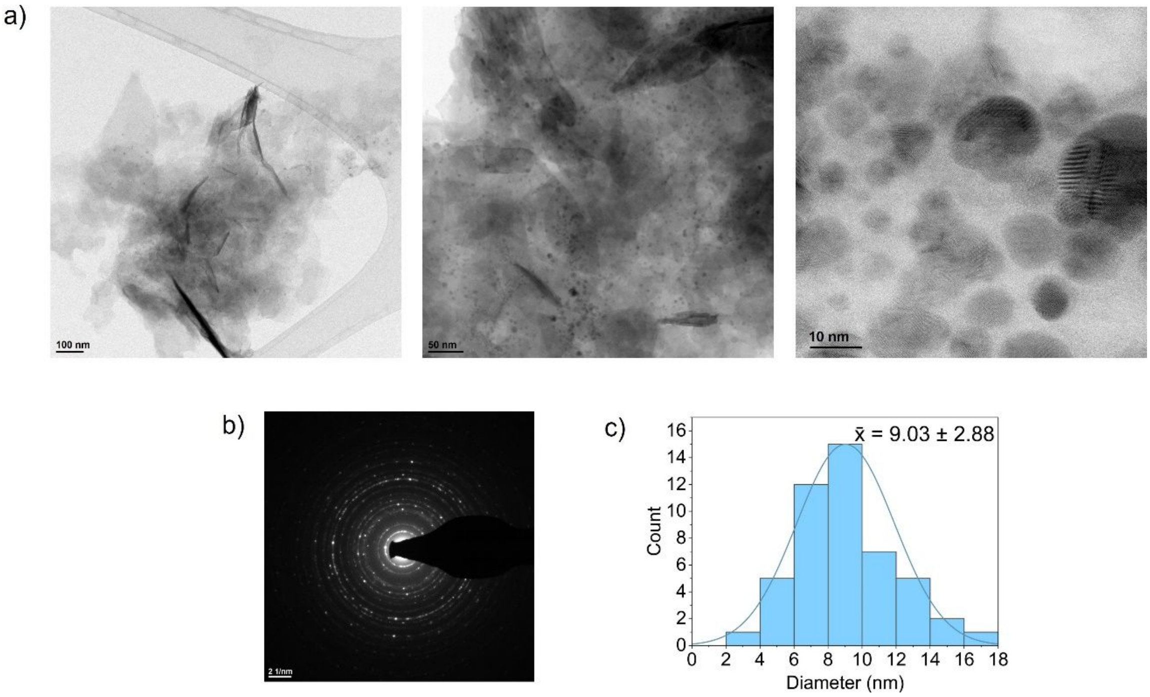

Figure S4. Characterization of Ag_2Cu_{34}@CALB bionanohybrid. A) TEM images. B) Fast Fourier transform (FFT) pattern.C) AgNPs size distribution.

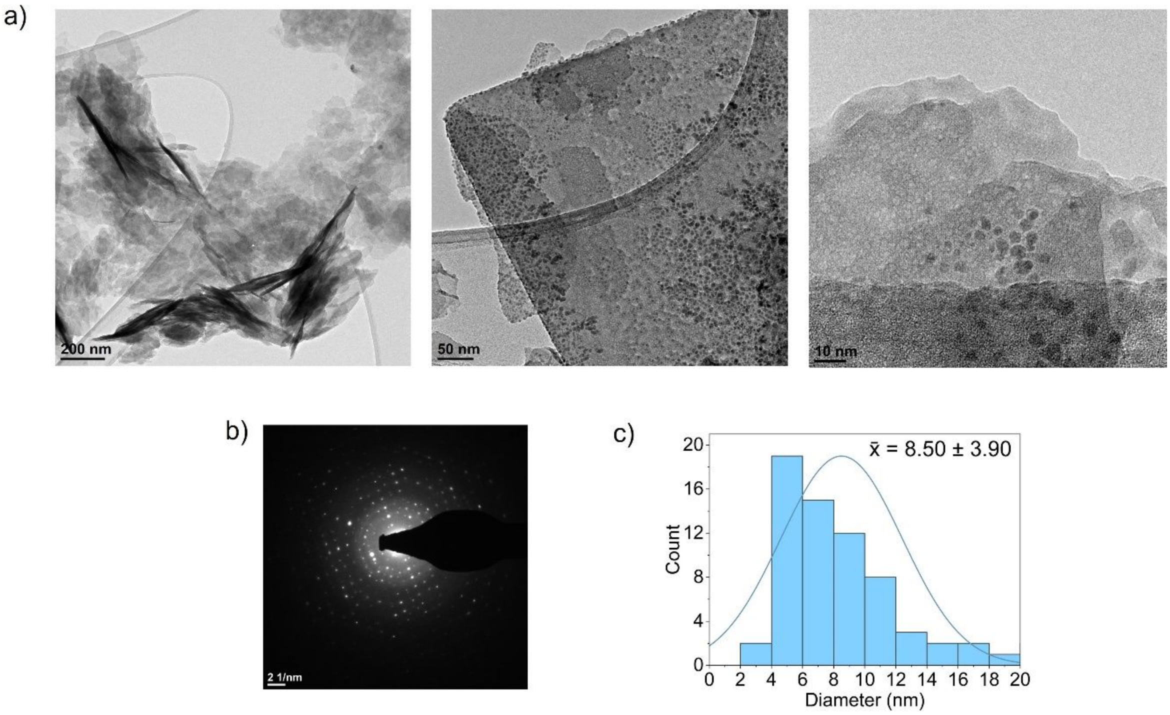

Figure S5. Characterization of Ag_4Cu_{32}@CALB bionanohybrid. A) TEM images. B) Fast Fourier transform (FFT) pattern.C) AgNPs size distribution.

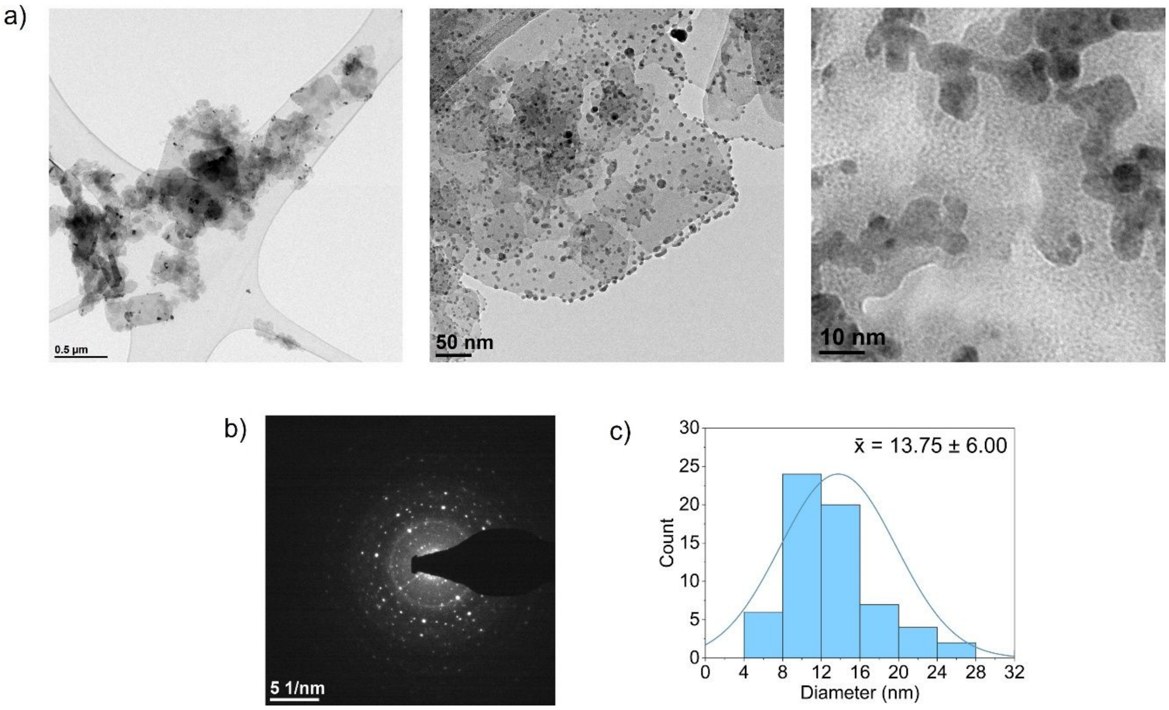

Figure S6. Characterization of Ag_8Cu_{28}@CALB bionanohybrid. A) TEM images. B) Fast Fourier transform (FFT) pattern.C) AgNPs size distribution.

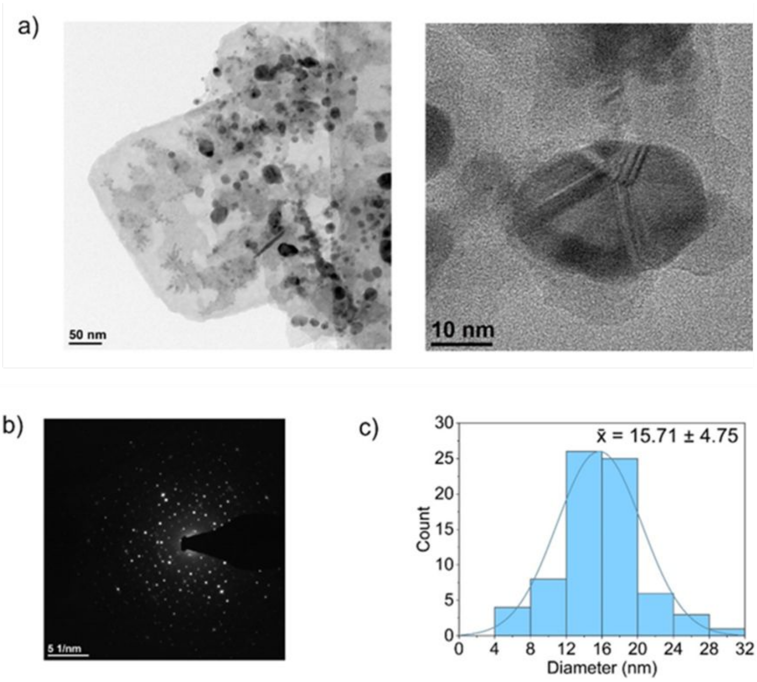

Figure S7. Characterization of $Ag_{15}Cu_{21}$@CALB bionanohybrid. A) TEM images. B) Fast Fourier transform (FFT) pattern.C) AgNPs size distribution.

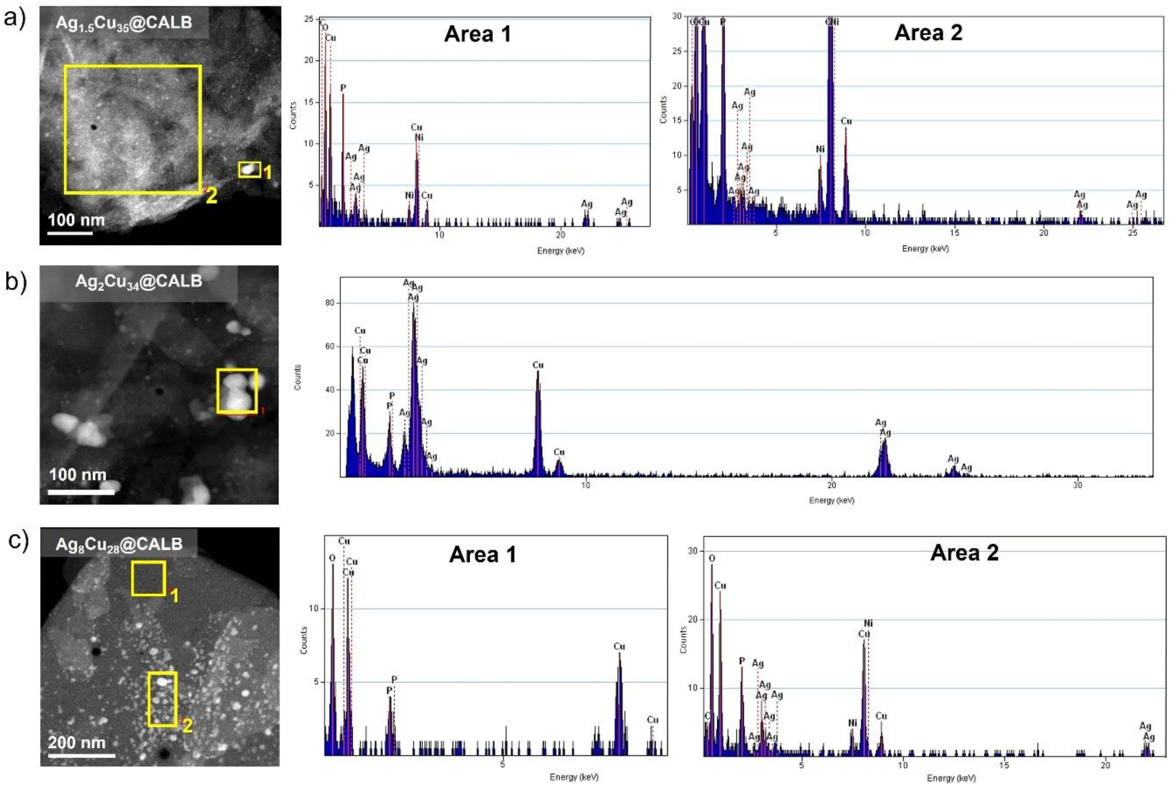

Figure S8. HAADF STEM imaging (left panels) and STEM-EDX analysis (right panels) of Cu and Ag nanoparticles. A) $Ag_{1.5}Cu_{35}$@CALB hybrid. B) Ag_2Cu_{34}@CALB hybrid. C) Ag_8Cu_{28}@CALB hybrid. The nickel signal corresponds to the grid used for the experiment.

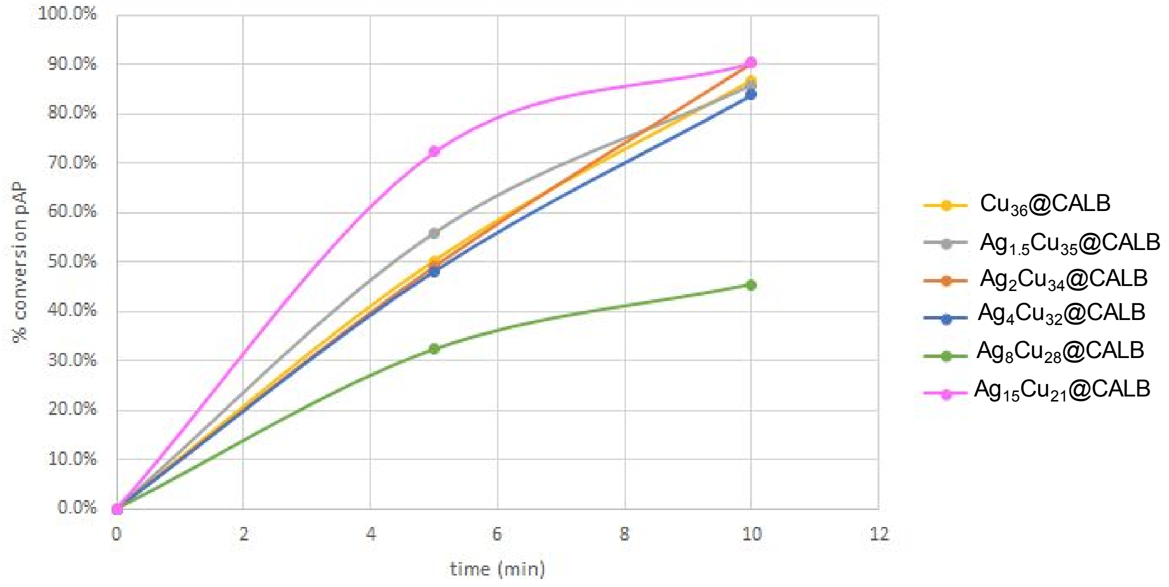

Figure S9. Profile of pAP oxidation reaction catalyzed by nanobiohybrids.

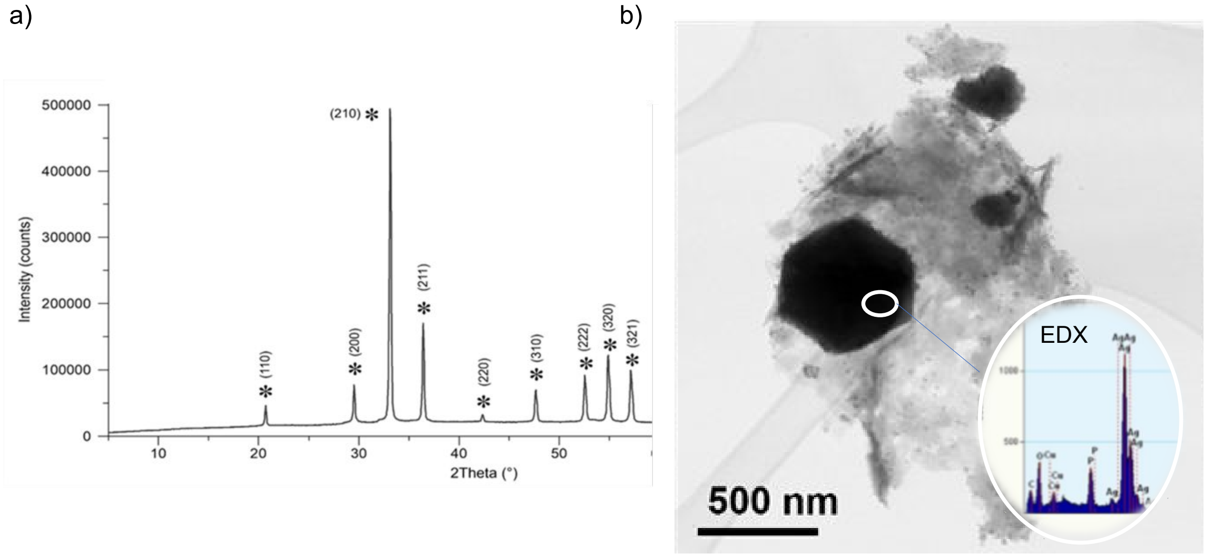

Figure S9. Characterization of $Ag_{32}Cu_8$@CALB hybrid. A) XRD pattern marked with * peaks corresponding to Ag_3PO_4. B) TEM image and STEM-EDX analysis (inside circle) of Ag nanoparticles.

a)

Hybrid	Ag in the hybrid (wt%)[a]	TOF [b] (min^{-1})
$Ag_{32}Cu_8$@CALB	31.7%	**0.44**
Ag_4Cu_{32}@CALB	3.7%	**1.01**

b)

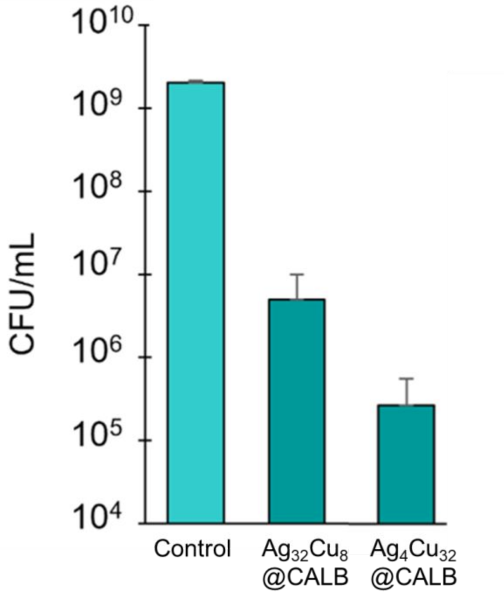

Figure S10. Comparison between Ag4 and Ag32 hybrid. A) TOF value determination in Fenton process in the oxidation of *p*-aminophenol. Reaction conditions: 1 mM pAP, 50 mM H_2O_2, 10 mL distilled water, 3 mg hybrid R.T.; [a]amount of silver calculated by ICP-OES, [b]TOF value was defined as the converted moles of 1 per moles of silver metal atoms in the bionanohybrid per minute calculated at conversion around 50%. B) Reduction in bacterial concentration (measured viable bacteria as CFU count per mL in the presence of the bionanohybrid relative to the control in the absence of the bionanohybrid) after a 4 h incubation with a concentration 125 ppm of each hybrid against E. coli. Graph shows the average of three different assays in CFU/mL. Error bars correspond to standard deviation.
